# Modification of Ischemia/Reperfusion-Induced Alterations in Subcellular Organelles by Ischemic Preconditioning

**DOI:** 10.3390/ijms23073425

**Published:** 2022-03-22

**Authors:** Paramjit S. Tappia, Anureet K. Shah, Bram Ramjiawan, Naranjan S. Dhalla

**Affiliations:** 1 Asper Clinical Research Institute, St. Boniface Hospital, Winnipeg, MB R2H 2A6, Canada; bramjiawan@sbrc.ca; 2 Department of Kinesiology, Nutrition and Food Science, California State University, Los Angeles, CA 90032, USA; akaur23@calstatela.edu; 3 St. Boniface Hospital Albrechtsen Research Centre, Department of Physiology and Pathophysiology, Institute of Cardiovascular Sciences, Max Rady College of Medicine, University of Manitoba, Winnipeg, MB R2H 2A6, Canada; ndhalla@sbrc.ca

**Keywords:** ischemia-reperfusion injury, subcellular remodeling, ischemic preconditioning, cardioprotection, oxidative stress, intracellular Ca^2+^-overload

## Abstract

It is now well established that ischemia/reperfusion (I/R) injury is associated with the compromised recovery of cardiac contractile function. Such an adverse effect of I/R injury in the heart is attributed to the development of oxidative stress and intracellular Ca^2+^-overload, which are known to induce remodeling of subcellular organelles such as sarcolemma, sarcoplasmic reticulum, mitochondria and myofibrils. However, repeated episodes of brief periods of ischemia followed by reperfusion or ischemic preconditioning (IP) have been shown to improve cardiac function and exert cardioprotective actions against the adverse effects of prolonged I/R injury. This protective action of IP in attenuating myocardial damage and subcellular remodeling is likely to be due to marked reductions in the occurrence of oxidative stress and intracellular Ca^2+^-overload in cardiomyocytes. In addition, the beneficial actions of IP have been attributed to the depression of proteolytic activities and inflammatory levels of cytokines as well as the activation of the nuclear factor erythroid factor 2-mediated signal transduction pathway. Accordingly, this review is intended to describe some of the changes in subcellular organelles, which are induced in cardiomyocytes by I/R for the occurrence of oxidative stress and intracellular Ca^2+^-overload and highlight some of the mechanisms for explaining the cardioprotective effects of IP.

## 1. Introduction

Although myocardial ischemia due to a partial or complete blockage of the coronary artery is known to induce marked changes in cardiac function and metabolism, as well as the ultrastructural integrity of cardiomyocytes [1,2], the pathophysiological mechanisms that lead to contractile dysfunction and derangement of cardiac structure are not fully understood. While coronary reflow to the ischemic heart is helpful for the recovery of contractile function, reperfusion after a certain period of prolonged ischemia exacerbates damage to the heart [3,4,5,6,7,8]. Two major abnormalities, namely intracellular Ca^2+^-overload and oxidative stress, are considered to play a critical role in cardiac contractile dysfunction subsequent to ischemia/reperfusion (I/R) or hypoxia–reoxygenation injury. In this regard, the beneficial effects of Ca^2+^-antagonists [9,10] and Na^+^-H^+^ exchange inhibitors [11,12,13,14] support the role of intracellular Ca^2+^-overload [15,16,17,18,19,20,21], whereas the beneficial effects of antioxidants [4,5] suggest the role of oxidative stress in the pathophysiology of ischemia–reperfused hearts. It is pointed out that alterations in the function of different subcellular organelles such as sarcolemma (SL), sarcoplasmic reticulum (SR), myofibrils and mitochondria due to I/R injury have been demonstrated to be associated with depression in cardiac contractile activity, the development of intracellular Ca^2+^-overload and the occurrence of oxidative stress [4,5]. It is also known that repeated episodes of brief ischemia followed by reperfusion, which is referred as ischemic preconditioning (IP), attenuates subcellular organelle remodeling due to subsequent I/R [22,23]. This review is designed to examine the information regarding the effects of IP on the development of oxidative stress and intracellular Ca^2+^-overload with respect to the modification of subcellular defects due to I/R injury [4,5]. In particular, some of the mechanisms of beneficial action of IP on molecular and functional changes for Ca^2+^-handling by subcellular organelles will be described in hearts subjected to I/R injury. Furthermore, the effects of IP on some factors, such as inflammatory cytokines and proteolytic activities, as well as the nuclear factor erythroid factor 2 (Nrf2) signal transduction pathway, which are known to modulate subcellular defects due to I/R injury, will be outlined. The generation of reactive oxygen species (ROS) and subsequent oxidative stress is known to increase apoptosis [24], necrosis [24,25], inflammation [24] and impair mitochondrial function [26,27]. In fact, IP has been demonstrated to attenuate the I/R-induced defects [28,29,30,31]. Thus, it can be seen that the generation of ROS has marked detrimental impacts on cardiomyocyte function and that IP may provide protection against oxidative stress. Therefore, the focus of this review is on the protective effects of IP on the I/R-induced defects in the heart.

## 2. Alterations in Cardiac SL and SR Ca^2+^-Transporting Activities Due to I/R

In order to understand the importance of the beneficial actions of IP with respect to Ca^2+^-homeostasis in cardiomyocytes, it is necessary to describe the impact of I/R on the Ca^2+^-handling properties of the two major subcellular organelles, namely SL and SR. It has become evident that ROS and other oxidant molecules are involved in the genesis of intracellular Ca^2+^-overload, myocardial cell damage and subsequent contractile dysfunction due to I/R injury [3,4,5,32]. Electron paramagnetic resonance spectroscopic studies have revealed the generation of ROS during reperfusion of the ischemic heart [33,34,35]. Dysregulation of cardiomyocyte Ca^2+^-movements in response to excessive ROS generation and the inability of cardiomyocytes to scavenge ROS by endogenous antioxidant systems (a state of oxidative stress) results in the development of intracellular Ca^2+^-overload subsequent to I/R injury [36,37,38,39]. Since SL Ca^2+^-channel density has been reported to decrease due to hypoxia/regeneration and myocardial ischemia [40,41], or when SL membranes are treated with oxyradicals [42], this mechanism cannot be seen to account for the occurrence of intracellular Ca^2+^-overload, as it would decrease Ca^2+^-influx. On the other hand, the activities of both SL Na^+^-Ca^2+^ exchange and the SL Ca^2+^-pump have been observed to be markedly reduced subsequent to hypoxia or I/R injury [43,44,45], as well as when heart membrane preparations are exposed to oxyradicals [46,47,48,49]; these alterations would decrease in Ca^2+^-efflux and have been suggested to produce intracellular Ca^2+^-overload. Other SL activities, such as Na^+^-K^+^-ATPase, Ca^2+^/Mg^2+^ ecto-ATPase and superficial store of Ca^2+^ [49,50,51,52,53,54,55,56,57,58,59] are also affected by ROS and oxidant molecules and are considered to increase Ca^2+^-influx in the cell indirectly. It should be noted that the actions of ROS as well as I/R injury on the SL Na^+^-Ca^2+^ exchange, Na^+^-K^+^-ATPase and Ca^2+^/Mg^2+^ ecto-ATPase seem to depend on the type of oxyradical generating system and the time of exposure to these interventions [37,48,49,60,61,62]. It should be noted that several other SL defects are also known to occur in hearts subjected to I/R or exposure of heart membranes to oxyradicals and oxidant molecules; these include SL Ca^2+^ transporters [63,64,65,66], ATP-receptors, β-adrenoceptors, G-proteins, adenylyl cyclase, phospholipases, as well as alterations in membrane permeability [67,68,69,70,71,72,73,74,75,76,77,78,79,80] and loss of dystrophin [81,82]. From these observations, it is evident that the increase in the formation of ROS and oxidants in ischemic hearts upon reperfusion may induce a number of different and complex defects in the SL membrane for modifying Ca^2+^-fluxes, but on balance would result in the development of intracellular Ca^2+^-overload in cardiomyocytes. It should also be mentioned that oxidative stress generated due to I/R injury has been shown to cause alterations in excitation–contraction coupling as well as induce cardiac arrhythmias, heart dysfunction and myocardial cell damage [83,84,85,86,87,88,89].

Changes in SR Ca^2+^- transporting activities are also known to occur under conditions of I/R as well as upon exposure of the heart to different oxidant molecules. In this regard, a reduced ability of cardiac SR Ca^2+^-uptake due to oxyradicals has been attributed to attenuation of Ca^2+^-stimulated ATPase activities [90,91,92]. A progressive loss of Ca^2+^-release channels has also been observed following exposure of the SR to ROS [93], but this may not represent the status of Ca^2+^-release from SR under the in vivo situations for oxidative stress. The rotenone-insensitive NADH cytochrome C reductase activity of the cardiac SR has also been reported to be reduced in response to oxyradicals and has been linked to SR lipid peroxidation [57]. Although ATP-dependent Ca^2+^-uptake and Ca^2+^-stimulated ATPase activities of skeletal muscle SR are depressed by ROS, this effect was suggested to be due to the inhibition of the sulfhydryl groups of the Ca^2+^-pump ATPase rather than membrane lipid peroxidation [94,95]. Interestingly, activated neutrophils, which are known to suppress the contractile force development of the heart [96], are considered to decrease the SR Ca^2+^-pump activities due to the generation of oxidants [97]. Overall, these alterations due to I/R injury can be seen to contribute to the occurrence of intracellular Ca^2+^-overload in cardiomyocytes. It is therefore clear that several subcellular Ca^2+^-handling proteins can be targeted as mechanisms for defects in SL and SR Ca^2+^-handling activities and inducing intracellular Ca^2+^-overload due to I/R injury. Furthermore, it should also be pointed out that cardiac dysfunction due to I/R injury is not limited to defects in SL and SR membranes but to other subcellular organelles such as the mitochondria, myofibrils and extracellular matrix, which are also affected in inducing myocardial abnormalities [4,5,20,21,22,23,32].

## 3. Subcellular Modification Due to Ischemic Preconditioning

It has been demonstrated that IP limits the infarct size, and attenuates necrosis, apoptosis as well as cardiac dysfunction due to I/R injury [98,99,100,101,102,103]. These beneficial effects of IP are reported to be mediated through the activation of adenosine receptors [99] and protein kinase C activity [101,104,105,106,107], as well as due to reduced phospholipase activities [108] and the attenuation of TNF-α levels [109].

**Figure 1:**
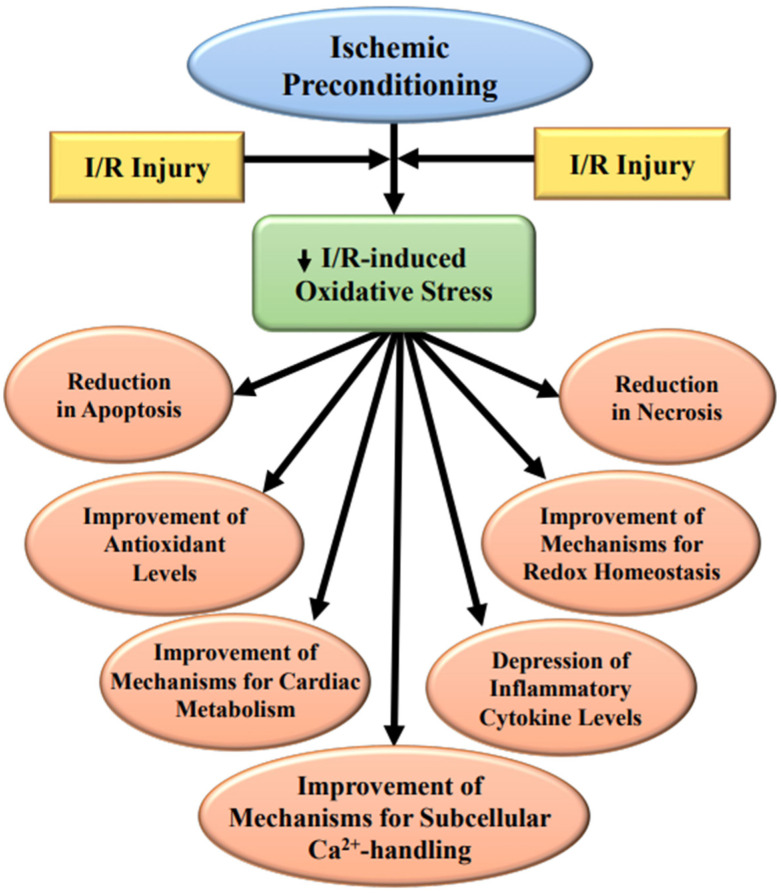
Alterations in some ischemia–reperfusion induced (I/R) parameters in hearts subjected to ischemic preconditioning.

The glycocalyx is particularly susceptible to I/R injury, as it is the first to be exposed to ROS, but IP has been shown to provide partial protection [110]. Furthermore, IP attenuates the cleavage of myofilament troponin I by matrix metalloprotease 2 (MMP-2) [111] upon decreasing its release and activation due to I/R injury. It is pointed out that the activation and release of MMP-2 are directly correlated with cardiac dysfunction due to I/R injury [111,112], and these alterations have been suggested to be due to the formation of hydroxy radicals and peroxynitrite, which have been shown to cause myofibril damage [113]. IP has also been observed to be protect the cytoskeleton from I/R-induced damage due to the activation of the ROS-p38 MAPK-HSP 27 pathway that initiates actin filament polymerization, as well as increases the stability of the contractile apparatus [114,115,116]. It is therefore evident that IP induces cardioprotective actions against the I/R-induced abnormalities by reducing the development of oxidative stress and associated defects. Some of the parameters affected by IP in ischemic–reperfused hearts are depicted in Figure 1.

### 3.1. Protection of the SL Defects

There are a variety of receptors and ion channels/pumps located in the SL membrane that are considered to influence cardiomyocyte function through the activation of downstream signal transduction processes and are affected by I/R injury, and thus may serve as targets for providing cardioprotection in IP. For example, the activation of several different receptor types, including adenosine receptors [117], angiotensin II receptors [102,118,119], α_1_- adrenoceptor [120,121] and opioid receptors [122,123], have been shown to be involved in the initiation of cardioprotective mechanisms in response to IP. During ischemia, there is an increase in the cardiomyocyte concentration of Na^+^ due to the exchange with protons via the activation of the SL Na^+^-H^+^ exchanger (NHE), which causes the activation of the SL Na^+^-Ca^2+^ exchanger in reverse mode, resulting in an increase in the intracellular Ca^2+^-concentration [124]; thus, the inhibition of NHE has been observed to attenuate intracellular Ca^2+^-overload [125]. The beneficial actions of IP have been suggested to involve a reduction in the activation of NHE.

The Na^+^-K^+^-ATPase (sodium pump) is known to play an important role in the maintenance of the membrane potential and cation transport across the SL membrane [126]; specifically, Na^+^-K^+^-ATPase activity directly maintains intracellular Na^+^ and K^+^ concentrations as well as indirectly maintains intracellular Ca^2+^-concentration [127]. The α subunit of Na^+^-K^+^-ATPase is the catalytic domain and occurs in three isoforms: α1 is the most abundant but with a lower affinity for cardiac glycosides, as compared to the α2 and α3 subunits [128], while the β-subunit (also three isoforms) confers SL localization and insertion into the SL membrane. I/R has been reported to diminish Na^+^-K^+^-ATPase activity [129] and contribute to cardiac dysfunction. Thus, Na^+^-K^+^-ATPase can be considered as a viable mechanism for cardioprotection due to IP. In this regard, a reduction of the intracellular Ca^2+^-concentration has been reported to be due to the IP-induced preservation of Na^+^-K^+^-ATPase activity in the I/R hearts [52]. It should be mentioned that the Na^+^-K^+^-ATPase is considered to play a signaling function [130,131,132,133,134] and may act as a receptor transducing humoral signal [134]. Since ouabain activates several pathways that are involved in cardioprotection by IP, it has been suggested that protection against I/R injury by IP may involve the activation of the Na^+^-K^+^-ATPase/cSrc receptor complex and the subsequent stimulation of the key signaling mediators [135,136]. The reduction of infarct size due to IP is also considered to be due to Na^+^-K^+^-ATPase-mediated opening of the cardiac SL K^+^-ATP channels [137] and the subsequent reduction on the intracellular Ca^2+^-concentration, although these effects have been suggested to involve the mitochondrial K^+^-ATP channels [138].

It is pointed out that cardiac SL K^+^-ATP channels and the mitochondrial K^+^-ATP channels have been extensively studied in the cardioprotective effects of IP. Although it is now generally believed that the mitochondrial K^+^-ATP channels play a more significant role in the beneficial effects of IP, the SL K^+^-ATP channels have increased in importance for inducing the harmful effects of oxidative stress [139]. Earlier observations have revealed that the beneficial actions of IP may involve the SL K^+^-ATP channels because they are in an open state when exposed to ROS and this correlated well with the cytoprotective properties [140,141,142,143]. Subsequent studies demonstrated that the SL K^+^-ATP channels appear to act as an effector of IP and facilitated in improving functional recovery. This proposition was evident when the cardioprotective effects induced by the activation of the SL K^+^-ATP were observed in the stress period, but not during IP. On the other hand, by using isolated adult rat cardiomyocytes and an isoflurane-induced protection technique, both SL and mitochondrial K^+^-ATP channels were observed to participate in the IP-induced cardioprotection [144].

It should also be mentioned that the beneficial actions of IP have been reported to be lost in a cardiac specific Kir 6.2 (the pore subunit of SL K^+^-ATP channels) knockout mouse model [145]. It has also been shown that activated SL K^+^-ATP channels are important in preventing cardiomyocyte apoptosis and mitochondrial damage during oxidative stress, as inhibition of SL K^+^-ATP channels promoted oxidative stress-induced apoptosis. Mitochondrial Ca^2+^-loading has been reported to be markedly increased upon the inhibition of SL K^+^-ATP channel in cultured HL-1 and neonatal cardiomyocytes [146]. Taken together, it is evident that reduction in oxidative stress-induced alterations of the SL membrane plays an important role in cardioprotection by IP.

### 3.2. Protection for the SR Defects

Under conditions of I/R and/or oxidative stress marked changes in SR Ca^2+^-transport are associated with increase in the cytosolic Ca^2+^ level and decreasing ATP causing damage to the cell [147,148,149,150]. Decreases in the SR Ca^2+^-cycling protein mRNA levels due to I/R have also been demonstrated; however, IP has been shown to attenuate these changes by preventing the development of intracellular Ca^2+^ overload and thereby maintaining SR function [151,152]. It should be mentioned that ROS exert direct effects on SERCA 2 (SR Ca^2+^-stimulated-ATPase) by decreasing its activity and causing an increase in the SL Na^+^-Ca^2+^ exchanger (NCX) activity. These observations indicate that redox-dependent SR Ca^2+^ depletion may be partly affected by a reciprocal regulation of SERCA 2 and NCX [153]. Furthermore, the modification of –SH groups under conditions of oxidative stress have also been shown to compromise Ca^2+^-stimulated ATPase activity [154]. On the other hand, the oxidation of the –SH groups of SR ryanodine receptors (RyRs) are an important mechanism of IP for attenuating the I/R-induced intracellular Ca^2+^-overload [155].

### 3.3. Protection of the Mitochondrial Defects

There is a wealth of information available in the literature regarding the role of mitochondrial alterations in IP-induced cardioprotection in I/R hearts. [37,156,157,158]. The mitochondria are of particular interest because of their involvement in cell apoptosis and ROS generation. The major mechanism of cardioprotection related to this organelle is the closing of mitochondrial permeability pores and subsequent maintenance of the inner membrane potential and energy production [15,159]. As already mentioned, the mitochondrial K^+^-ATP channel acts not only as an effecter of IP, but also as a trigger, suggesting that its activation is required for both IP as well as during exposure to oxidative stress [153]. Of note, unlike the SL K^+^-ATP channel, the mitochondrial K^+^-ATP channel has been reported to primarily have an effect on the infarct size [153]. Pharmacological preconditioning of the rat heart with diazoxide results in the opening of the mitochondrial K^+^-ATP channel, which protects against ischemia-induced ventricular arrhythmias [160]. ROS released from the mitochondria during IP causes the opening of the mitochondrial K^+^-ATP channels, which is protective [161]. It is thus apparent that mitochondrial K^+^-ATP channels play an important role in IP-induced cardioprotection against I/R injury. On the other hand, donors of nitroxyl (the one-electron reduced form of nitric oxide) have been shown to provide protection against I/R injury in a manner similar to IP [162], with the translocation of PKCε to the mitochondria, but without the activation of the mitochondrial K^+^-ATP channels [163].

During IP, the mitochondria also undergo other modifications. In this regard, IP increases the expression level of manganese superoxide dismutase (MnSOD) via the formation of NFκB and activator protein-1 (AP-1) [164]. The increased production of oxyradicals may impair mitochondrial function, as an increase in the production of ATP has been observed in preconditioned hearts [165]. Although the ROS-induced opening of the mitochondrial permeability transition pore (mPTP) results in a further release of ROS [5,166,167], IP prevents the mPTP from opening and attenuates the burst of the ROS and the subsequent oxidative stress [5,166]. It has also been suggested that IP may cause a gradual activation of mitochondrial function that may avoid the ROS bursts and occurrence of mitochondrial Ca^2+^ overload [168].

It needs to be emphasized that complex I (NADH ubiquinone oxidoreductase) is the point of entry of the electron into the mitochondria and is a major site of ROS generation [168,169]. An increase in the NADH/NAD+ redox balance has been shown to inhibit the opening of the mPTP [169,170]. IP and NO∙ have both been observed to inhibit complex I activity and thus diminish ROS generation [169]. Interestingly, the inhibition of complex II has been reported to open the mitochondrial K^+^-ATP channel and result in cardioprotection [169,171]. Glyceraldehyde 3-phosphate dehydrogenase has also been observed to be inhibited by IP [172,173]. This action results in the accumulation of fructose-1,6-bisphosphate, which has been reported to improve glycolytic flux and functional recovery in post-ischemic myocardium [174]. Furthermore, lactate, which is known to accumulate in the ischemic heart, was observed to be markedly reduced in IP [174]. These findings demonstrated that IP can inhibit glycolysis and thereby prevent acidosis [169]. It should also be mentioned that the translocation of hexokinase from the cytosol to the mitochondrial compartment has been shown to occur in IP, which attenuates cytochrome *c* release and ROS production [175,176]. Overall, it is becoming clear that oxidative stress generated by the mitochondria may result in mitochondrial Ca^2+^-overload and the impairing of their function. These mitochondrial defects are attenuated by IP through the participation of diverse mechanisms.

### 3.4. Evidence for Attenuation of I/R-Induced SL and SR Defects by IP

While the aforementioned has described the IP-induced cardioprotection of subcellular organelle defects due to I/R, we have earlier examined some of the SL and SR mechanisms for cardioprotection induced by IP. Specifically, the effects of IP on Na^+^-K^+^-ATPase mRNA levels, protein contents and activity have been reported. Based on the analysis of information in our paper [52], while I/R attenuated Na^+^-K^+^-ATPase mRNA and protein levels as well as the activity, IP reduced the I/R-induced changes, demonstrating that cardioprotection due to IP involves maintenance of Na^+^-K^+^-ATPase. Table 1 shows the effect of IP (three cycles of 10 min ischemia/10 min reperfusion) on Na^+^-K^+^-ATPase activity and Na^+^-K^+^-ATPase isoform (α1, α2, α3) protein contents and mRNA levels in the I/R hearts [52]. In addition, we also explored the effects of IP on the Ca^2+^-handling properties of hearts subjected to a prolonged period of I/R. From the data in Table 2 showing SR Ca^2+^-release and Ca^2+^-uptake activities, as well as SR Ca^2+^-stimulated ATPase activity in preischemic, ischemic and ischemic–reperfused control and preconditioned hearts, it has been suggested that IP prevents cardiac contractile dysfunction by protecting SR Ca^2+^ movements in cardiomyocytes [151]. In addition, the protective effect of IP on SR gene expression in the I/R hearts is shown in Table 3 [152]. Based on the information in our papers [151,152], it is thus also evident that the beneficial actions of IP have been attributed, in part, to protecting SR Ca^2+^ movements by reducing the development of I/R-induced oxidative stress in cardiomyocytes. These earlier findings provide evidence that an attenuation of the I/R-induced defects in both the SL and the SR can be considered as important mechanisms for the beneficial actions of IP-induced cardioprotection.

**Figure 2:**
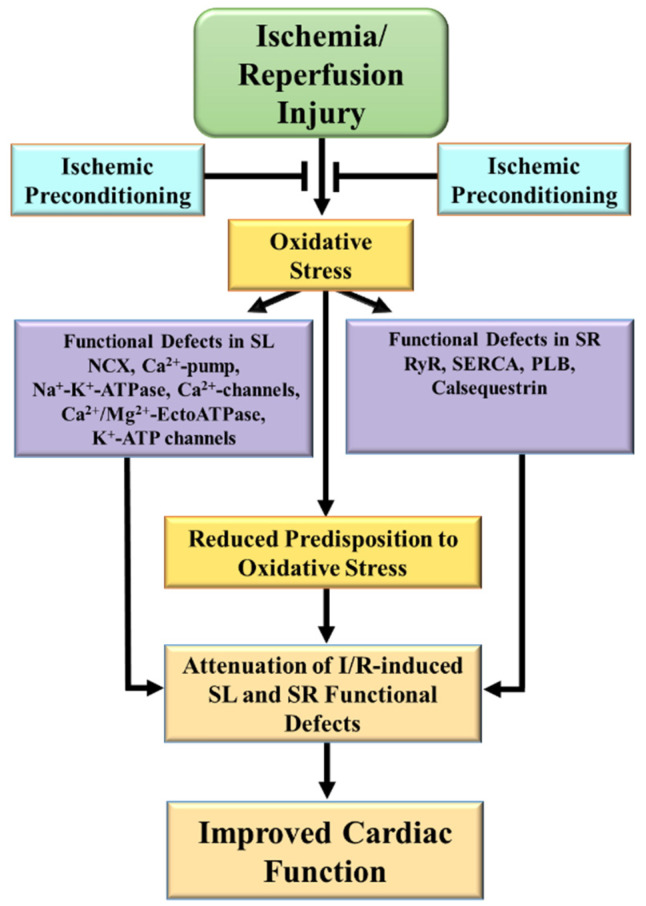
Functional defects the sarcolemma and sarcoplasmic reticulum membrane compartments due to ischemia/reperfusion and protective effects of ischemic preconditioning. SL = sarcolemma; SR = sarcoplasmic reticulum; NCX = Na^+^-Ca^2+^-exchanger; RyR = ryanodine receptor, SERCA = sarcoendoplasmic reticulum calcium transport ATPase; PLB = phospholamban; I–R = ischemia–reperfusion.

It should be pointed out that the expression and activities of different SL and SR proteins are altered in I/R, which are attenuated by IP [22,23,78,79,108,177], some of which are presented in Table 1 and Table 3. Figure 2 summarizes the functional defects of the SL and SR due to the oxidative stress induced by I/R, and that IP attenuates these defects associated with improved recovery of cardiac function.

## 4. Modification of I/R-Induced Defects in Signal Transduction

Reperfusion of the ischemic heart is vital to restore cardiac function, reduce infarct size and salvage the viable myocardium [127]. From the aforementioned, it is evident that cardiac dysfunction due to I/R injury is multifaceted and complex and that there are several different elements that can modulate intracellular Ca^2+^-concentration and subsequent I/R injury. Some of these factors include the production of inflammatory cytokines, the activation of proteases as well as Nrf2 signal transduction. By virtue of their ability to influence intracellular Ca^2+^-concentration, it is possible that an attenuation of the I/R-induced increases in inflammatory cytokines and proteolytic activity, as well as the regulation of Nrf2 signaling processes, may serve as important mechanisms in IP-induced cardioprotection. These aspects are briefly described in the following sections.

### 4.1. Role of Inflammatory Cytokines in IP-Induced Cardioprotection

The inflammatory cytokines, TNF-α, IL-1 and IL-6, are known to influence cardiomyocyte Ca^2+^-handling and subsequent cardiac dysfunction in I/R injury [178,179,180,181], and thus may be important targets/mechanisms for cardioprotection due to IP. Both myocardial macrophages and cardiomyocytes produce TNFα, which depresses the heart function and induces cardiomyocyte apoptosis [182]. It is now established that the inflammatory cytokines mimic IP when given as a single episode before prolonged ischemia [183]; indeed, inflammatory cytokines may induce late preconditioning [184]. Late IP of the myocardium has been reported to alter the expression of genes that are involved in the inflammatory response. In this regard, not only is the gene for MMP9 upregulated, but also the gene for TNFα is upregulated in the late phase of IP [185]. Interestingly, the classic IP antagonist, 5-hydroxydecanoate, has been reported to abolish TNFα-mediated IP [186]. IP has been shown to decrease postischemic myocardial TNFα, which may represent the distal effector mechanism of preconditioning [187]. The influence of IP on TNFα and cardioprotection has been further demonstrated by the observation that the IP-mediated reduction in infarct size is associated with a reduction in circulating TNFα levels in a rat model of I–R injury [188].

It is pointed out that while the adverse effects of IL-1 in I–R are known to be due to the activation of superoxide dismutase and cardiac remodeling, IL-1 has been shown to be cardioprotective in IP [189]. On the other hand, IL-6 is a pleiotropic cytokine that protects against cardiac I/R injury following pharmacological preconditioning and IP [190,191,192]. The beneficial actions of IL-6 in IP have been suggested to be mediated through an IL-6/STAT3-dependent mechanism [193]; in fact, IL-6-mediated late preconditioning via JAK–STAT signaling results in the upregulation of iNOS and COX-2 and the development of cardioprotection [194]. In addition, IL-6 has also been reported to protect mitochondrial energetics and function [181].

### 4.2. Impact of IP on the Activation of Proteolysis

Proteases play an important role in the degradation of misfolded or malfunctional proteins, as well as in the routine turnover of the extracellular matrix and other subcellular organelles, and thus are essential in the maintenance of cell homeostasis [3,195,196]. Proteases are basally active, and their activity is regulated through a number of factors including their transcription, translation, chaperone molecules and the presence of endogenous inhibitors. However, in different pathophysiological conditions, including I/R, there is a dysregulation of proteases that results in a substantial increase in the activities of several effector proteases, including calpain, matrix metalloproteinases (MMP) and cathepsins [195]. Since these proteases are significantly altered in I/R, it is thus likely that IP may exert beneficial effects with respect to an attenuation of their highly activated state [127]. In this regard, IP, which is known to attenuate oxidative stress as well as intracellular Ca^2+^-overload has also been reported to diminish the I/R-induced activation of calpain and MMP [197]. Thus, it can be suggested that oxidative stress, either directly or indirectly as a consequence of intracellular Ca^2+^-overload, may both play a key role in the activation of proteolytic activities subsequent to I/R [197]. It should be mentioned that LonP1 is considered an essential mitochondrial protease that is critical for the maintenance of mitochondrial proteostasis and protection against cell stress [198]. Interestingly, IP was shown to induce a twofold increase in LonP1, which protected the heart against I/R injury, and it was thus suggested that the upregulation of LonP1 mitigated cardiac injury by preventing oxidative damage as well as through preserving mitochondrial redox balance [198].

### 4.3. Role of Nrf2 Signal Transduction in IP-Induced Cardioprotection

Nrf2 is a transcription factor that controls cellular defense responses [199], particularly in the regulation of the expression of antioxidant and detoxification genes [199,200,201]. Nrf2 is known to protect cardiomyocytes against oxidative stress by increasing endogenous antioxidants [202,203]. Indeed, the Nrf2/antioxidant response element plays a central role in the protective effect against oxidative and apoptotic damage [204,205]. Specifically, as a transcription factor, Nrf2 triggers the production of many phase II detoxifying and antioxidant enzymes through the activation of heme-oxygenase 1 and NADPH quinone oxidoreductase gene expression, which protect cells under conditions of oxidative stress [206,207,208] and provides cellular protection against different toxins [206,209,210,211]. In fact, several studies have shown that the activation of Nrf2 and the subsequent upregulation of antioxidant enzyme gene expression is an important mechanism of cardioprotection due to ischemic/hypoxic preconditioning, and that low concentrations of ROS may be an essential signal for the protective process [212,213,214,215]. Furthermore, Nrf2 has also been reported to modulate intracellular Ca^2+^ levels [216], and thus cardioprotection against I/R injury due to the activation of Nrf2 has recently been explored for clinical applicability [199]. In addition, the role of Nrf2 signal transduction in the protective actions of IP has also been examined [212,217]. Indeed, the Nrf2/antioxidant response element pathway activation may be of importance in IP [218]. In Nrf2 knockout mice, an increase in infarct size in response to I/R has been reported, as well as a reduced cardioprotective effect of IP [219]. The activation of PKC is known to be triggered by IP [219]. In this regard, polymyxin B, an inhibitor of PKC, blocked the membrane translocation of PKCδ and PKCε during IP that was also associated with an inhibition of Nrf2 nuclear accumulation, as well as a reduction in the cardioprotective effects of IP [219]. It was thus suggested that the activation of PKC induces the translocation of Nrf2 and the upregulation of endogenous antioxidant defenses in IP, and that specific PKC isoforms may target Nrf2 to exert cardioprotection [219].

## 5. Conclusions

**Figure 3:**
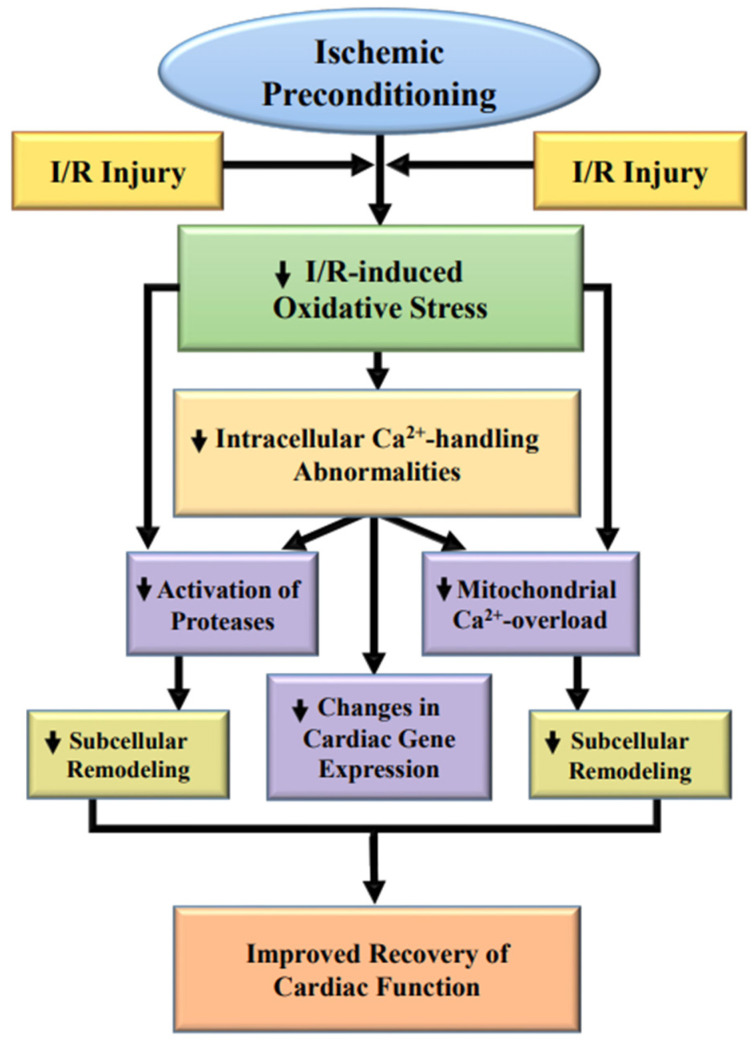
Proposed cardioprotective mechanisms of ischemic preconditioning for improving cardiac function in hearts subjected to ischemia/reperfusion (I/R) injury.

From the foregoing discussion, it is evident that IP-induced cardioprotection is associated with the reduction of I/R-induced oxidative stress and the subsequent depression in the development of intracellular Ca^2+^-overload in the I/R heart. A schematic representation of some critical events in eliciting improved cardiac function due to IP in the I/R hearts is given in Figure 3. It is also evident that I–R induces a wide variety of defects in subcellular organelles, both at the functional and molecular level. Although oxidative stress can be seen to produce adverse effects on subcellular organelles or subcellular remodeling through the development of intracellular Ca^2+^-overload, oxidative stress can directly cause subcellular and molecular abnormalities. While the initial functional changes occur rapidly under conditions of oxidative stress, the delay in functional recovery of the ischemic heart upon reperfusion may be due to alterations in the gene expression levels of SL and SR proteins, including those for other subcellular organelles such as the mitochondria, myofibrils and extracellular matrix. On the other hand, IP attenuates the occurrence of intracellular Ca^2+^-overload and oxidative stress in the I/R hearts, and thus reduces the I/R-induced subcellular remodeling and cardiac dysfunction. In addition, attenuation of proteolysis, but activation of Nrf2-mediated signal transduction, can also be considered as one of the important mechanisms of beneficial action of IP-induced cardioprotection. However, the translation of experimental interventions to clinical therapies remains to be a challenge.

## Figures and Tables

**Table 1:** Effect of ischemic preconditioning on Na^+^-K^+^-ATPase activities, protein contents and mRNA levels.

	Control	IP	I/R	IP + I/R
Na^+^-K^+^-ATPase activity (µmol Pi/mg/h)	15.0 ± 0.6	14.8 ± 0.8	11.3 ± 0.7 *	15.4 ± 0.7 ^#^
Protein content (arbitrary units)				
Na^+^-K^+^-ATPase α1	15.7 ± 1.3	15.0 ± 2.1	11.2 ± 1.4 *	13.2 ± 1.8
Na^+^-K^+^-ATPase α2	7.3 ± 1.8	7.1 ± 1.3	2.1 ± 1.5 *	6.2 ± 1.1 ^#^
Na^+^-K^+^-ATPase α3	2.3 ± 0.2	2.2 ± 0.5	0.8 ± 0.4 *	1.4 ± 0.5 ^#^
mRNA expression levels (% of control arbitrary units)				
Na^+^-K^+^-ATPase α1	100	140 ± 11 *	79 ± 3 *	95 ± 2
Na^+^-K^+^-ATPase α2	100	90 ± 5	51 ± 4 *	78 ± 4 ^#^
Na^+^-K^+^-ATPase α3	100	100 ± 20	32 ± 5 *	61 ± 12 ^#^

Ischemic preconditioning was 3 cycles of 10 min ischemia/10 min reperfusion. Values for Na^+^-K^+^-ATPase activity are means ± 12–18 hearts in each group. For Western blot determination of protein contents, each protein sample was a collection of three hearts. Northern blot analysis was conducted using 18S mRNA level as internal standard and each value is the mean ±SE of 6 hearts/group. Control value is taken as 100% for each gene under different condition. * *p* < 0.05 vs. control. ^#^ *p* < 0.05 vs. I–R. Data are based on the analysis of information in our paper Elmoselhi et al. [52]. IP = ischemic preconditioning; I/R = ischemia/reperfusion.

**Table 2:** SR Ca^2+^-release and -uptake activities and SR Ca^2+^-stimulated ATPase activity in preischemic, ischemic and ischemic–reperfused control and preconditioned hearts.

SR Ca^2+^-Release (nmol/mg/15 s)	Preischemia	Postischemia	Reperfusion
Control	17.5 ± 5.1	2.0 ± 0.5 ^#^	2.5 ± 0.5 ^#^
Precondition	6.7 ± 5.2 *	7.3 ± 2.2 *	11.2 ± 4.5 *
SR Ca^2+^-uptake (nmol/mg/min)			
Control	70.8 ± 8.1	8.0 ± 1.1 ^#^	9.5 ± 2.0 ^#^
Precondition	38.6 ± 6.4 *	28.3 ± 3.7 *	24.5 ± 1.5 *
SR Ca^2+^-stimulated ATPase activity (nmol Pi/mg/min)			
Control	199.8 ± 28.4	102.0 ± 20.4 ^@^	65.5 ± 13.4 ^@^
Precondition	201.4 ± 16.9	203.5 ± 26.4 ^!^	146.1 ± 19.6 ^!^

The SR Ca^2+^-release is the EGTA-induced Ca^2+^-release from Ca^2+^-loaded SR vesicles; Ca^2+^-uptake is the oxalate-supported Ca^2+^-uptake in SR vesicles in the presence of ruthenium red. Values are means ±SE of 6 different preparations for each time point for each group. * *p* < 0.05 vs. corresponding control value. ^#^ *p* < 0.05 vs. preischemic group. Ca^2+^-stimulated ATPase activity was calculated from the difference between values in the presence and absence of 10 µM Ca^2+^. ^!^ *p* < 005 compared with respective control value. ^@^ *p* < 0.05 compared with preischemic control value. Data are based on the analysis of information in our paper Osada et al. [151].

**Table 3:** Effect of ischemic preconditioning on SR gene expression and relative protein contents in the I–R hearts.

A: mRNA Expression Levels (% of Control Arbitrary Units)	IP	I/R	IP + I/R
RyR	68.2 ± 4.4 *	35.7 ± 4.2 *	72.1 ± 4.7 *^,#^
SERCA	76.9 ± 4.9 *	70.8 ± 2.4 *	90.3 ± 3.4 *^,#^
PLB	74.1 ± 3.2 *	40.6 ± 3.6 *	67.5 ± 4.8 *^,#^
Calsequestrin	100 ± 3.5	50.8 ± 4.5 *	71.8 ± 6.4 *^,#^
**B: Relative protein content** **(% of control arbitrary units)**			
RyR	73.4 ± 8.2 *	51.7 ± 9.1 ^#^	923 ± 8.6 *
SERCA	101.1 ± 18.1	45.4 ± 2.2 ^#^	81.2 ± 17.1 *
PLB	118.1 ± 19.2	100.3 ± 18.3	105.5 ± 19.4
Calsequestrin	n.d.	n.d.	n.d.

Ischemic preconditioning was 3 cycles of 5 min ischemia/5 min reperfusion. (A) Northern blot analysis was conducted using 18S mRNA level as internal standard. (B) Determination of protein contents was conducted by Western blot analysis. Each value is the mean ± SE of 6 hearts/group in the case of mRNA analysis and 6 different SR preparations in the case of analysis of protein contents. Values are expressed as a percentage of control values. Control value is taken as 100% for each gene/protein under different condition. * *p* < 0.05 vs. control. ^#^ *p* < 0.05 vs. I–R. Data are based on the analysis of information in our papers Osada et al. [151] and Temsah et al. [152]. IP = ischemic preconditioning; I/R = ischemia/reperfusion; RyR = ryanodine receptor; SERCA = sarcoendoplasmic reticulum calcium transport ATPase; PLB = phospholamban; n.d., not determined.

## Data Availability

Not applicable.
